# The Facilitation of Cognitive Procedures is not Dependent on Maintenance of Declarative Memory Elements

**DOI:** 10.5964/ejop.17457

**Published:** 2026-08-28

**Authors:** Michael F. S. Baranski, Daniel Byrnes, Katelyn L. McClure, Phillip Hamrick, Christopher A. Was

**Affiliations:** 1 Department of Psychology, Counseling, and Art Therapy, PennWest University, California, PA, USA; 2 Department of Psychological Sciences, Kent State University, Kent, OH, USA; Lancaster University, Lancaster, United Kingdom

**Keywords:** declarative memory, procedural memory, facilitation of cognitive procedures

## Abstract

Previous research has demonstrated that the cognitive procedure of categorization can be facilitated with practice. That is, practice categorizing exemplars of a particular category results in faster and more accurate categorizing of new exemplars within processed categories compared to non-processed categories. Further, subsequent research using a directed-forgetting paradigm demonstrated that this facilitation of procedural memory can occur without the maintenance of declarative memory elements (i.e., category exemplars). The current research extends these findings by examining if procedural memory can be facilitated in the absence of declarative memory recall of processed category labels. Results demonstrate another replication of the facilitation of procedural memory as well as non-recalled category exemplars being categorized faster and more accurately than non-processed category exemplars. Interestingly, participants more quickly and accurately categorized non-recalled categories compared to recalled categories. The results suggest that the facilitation of exemplar comparisons is not dependent upon category label maintenance in declarative memory.

Previous research has demonstrated that the cognitive procedure of categorization can be facilitated with practice (e.g., Woltz & Was, 2006, 2007). That is, practice categorizing exemplars of a particular category results in faster and more accurate categorizing of new exemplars within this practiced category compared to unpracticed categories. The current study was designed to (1) replicate and extend research on the facilitation of procedural memory and (2) test the hypothesis that durable facilitation of specific cognitive operations, such as categorization, is not reliant on the active maintenance of exemplars in declarative memory elements.

## Facilitation of Procedural Memory

Woltz and Was (2006, 2007; see also Was, 2010; Was & Woltz, 2007) demonstrated the facilitation of specific cognitive operations following simple processing in working memory. The facilitation of procedural memory occurred when participants were first required to commit to memory a list of four words (i.e., memory load; e.g., *oak, table, elm, chair*) comprised of two exemplars chosen from two categories. In this case *trees* and *furniture.* One of the categories was explicitly named in the instructions (e.g., “*remember the trees*”), and participants later recalled exemplars from this *remember* category. Following the recall of the two specified exemplars, participants completed a series of category comparison trials. In the comparison trials, participants indicated whether two presented words came from the same or different categories. Participants were presented with an equal number of trials in which the two exemplars came from the same and different categories. They were also presented with an equal number of category comparisons in which the two exemplars were new examples of items from the remember category, the ignore category, and a novel category not represented in the memory load. For example, participants might be presented with *pine* and *willow*, representing two words from the same category, or *desk* and *truck* representing two words from different categories. Responses were faster and more accurate for pairs of associates from the remember category compared to the category in the memory load not committed to memory (i.e., *ignore* category) as well as the novel *unprimed* category. Responses to category comparisons of associates from the ignore category were also faster and more accurate than responses to category comparisons of associates from the unprimed category.

Regarding the greater facilitation for the remember category exemplars compared to ignore and unprimed category exemplars, Woltz and Was (2006) proposed two possible explanations. First, the extra maintenance of the remember exemplars required for later recall led to the greater facilitation. Second, the explicit naming of the remember category led to greater facilitation of this category and its exemplars. To test these alternative hypotheses, Woltz and Was (2006) conducted a second experiment during which they alternated the remember instruction with an ignore instruction. In half of the trials, immediately after the memory load, participants were instructed to remember the two exemplars from one category (e.g., “remember the trees”). On the other half of the trials, participants were instructed to forget exemplars from a category (e.g., “ignore the fruit”).

Woltz and Was (2006; Experiment 2) found that the magnitude of facilitation was greatest for the explicitly named category following the memory load. Put differently, responses to the category comparisons associated with the explicitly named category, regardless of whether they were from the remember or ignore category, were faster and more accurate than novel unprimed category comparisons. More importantly, facilitation for the explicitly named category (e.g. ignore) was greater than the facilitation for the other category (e.g., remember) in the memory load. This was the case even though following both instructions, participants were only required to later recall the exemplars of the remember category.

These results suggest that, for the demonstrated facilitation effect, the explicit category naming was more important than maintenance and recall of the to-be-remembered items. Thus, the cognitive procedure of identifying the exemplars of categories to be remembered or to be ignored led to greater facilitation of that category. Woltz and Was (2006) conducted a third experiment in which participants were instructed to recall the to-be-remembered items at the end of each trial. That is, the trial sequence of Experiment 3 was memory load, remember/ignore instruction, category comparison phase, and, last, recall of the to-be remembered items. Moving the recall portion of the trial to the end required participants to maintain the to-be-remembered items longer. The hypothesis was that extended maintenance of the to-be-remembered items may lead to greater facilitation of the remember category comparisons. This was not the case. The results of Experiment 3 replicated those of Experiment 2 in that the magnitude of facilitation was greatest for the explicitly named category regardless of extended maintenance of the to-be-remembered items. It is important to note that there was no significant difference in recall accuracy between Experiments 2 and 3. The results indicated that maintaining items from the to-be-remembered category in declarative memory did not increase the facilitation of procedural memory effect relative to explicit naming of the category.

It seems clear that the explicit naming of a category led to greater facilitation of the category comparison procedure than did maintenance and recall of category exemplars. However, Woltz and Was (2006, 2007) only required participants to recall the to-be-remembered exemplars; they did not test participants’ memory for the ignore category exemplars. Therefore, it is unknown if participants would have been able to recall the ignored items as well as they did the remember items. It is possible that the ignored items were maintained along with the remember items. The results may not have indicated an effect of active memory maintenance because all the items (i.e., remember and ignore) were being maintained. Put differently, maintenance of items in declarative memory may have supported the observed facilitation of procedural memory.

## Facilitation of Procedural Memory Despite Dissociation With Declarative Memory

Hirsch et al. (2021) demonstrated over three experiments that the facilitation of procedural memory can be dissociated from declarative memory maintenance. In each experiment, a directed forgetting procedure was used during the memory load instruction to (a) present the category label for all facilitated categories and (b) to encourage forgetting of category exemplars (Experiments 1 and 2) and category labels (Experiment 3). Across all experiments, participants showed the typical facilitation of procedural memory effect. That is, participants were faster and more accurate at making category comparison judgments for exemplars from categories included in the memory load compared to novel unprimed categories. This facilitation occurred even for categories in which participants showed evidence of directed forgetting. That is, participants had lower recognition accuracy (i.e., higher rates of forgetting) for exemplars or category labels they were instructed to forget, suggesting that these declarative memory items (i.e., category exemplars, category labels) were not maintained during the task. Yet, participants showed facilitated procedural memory for categorizing within both remember and forget categories. As in Woltz and Was (2006), explicit naming of category labels led to the facilitation of procedural memory. In Hirsch et al. (2021) it was demonstrated that the facilitation of procedural memory was not dependent on declarative maintenance of category exemplars or category labels, as procedural memory was still facilitated for forgotten declarative exemplars and category labels.

The findings of Hirsch et al. (2021) extend those of Anderson (2009), Oberauer (2009), and Squire (1992). These authors all posit a distinction between declarative and procedural memory and their respective processes. Anderson’s (2009) Adaptive Control of Thought-Rational (ACT-R) model of cognition posited that goal states and active declarative memory elements trigger operations of procedural memory; these operations produce appropriate responses to move towards the goal state. Once procedural memory performs its operations, new declarative memory elements are activated, and the process repeats until the goal state is attained. Oberauer (2009) posited a similar distinction between declarative and procedural working memories; procedural working memory operations are initiated by and operate on current declarative working memory elements. Squire (1992) provided neuropsychological evidence from amnesic patients that procedural operations can be learned in the absence of new declarative memories. In sum, evidence at the behavioral, computational, theoretical, and neuropsychological (Poldrack et al., 2001) levels all suggest a distinction between declarative and procedural memory systems and their operations.

Hirsch et al. (2021) provided evidence that the facilitation of procedural memory does not depend on declarative memory maintenance. Hirsch et al. stated, “therefore, although the declarative representations of the problem are active during the categorization procedures, continued maintenance of declarative activation is not necessary for the efficient reuse of the procedure. The procedure remains facilitated regardless of whether the relevant declarative nodes have become inactive” (p. 57). However, given the procedure in Hirsch et al. (i.e., category comparisons followed by recognition task), it is possible that declarative elements (e.g., category labels) could be active and maintained during the facilitation of procedural memory and thereby support this facilitation, but lose activation and cease to be maintained by the recognition task. The aim of this study was to test this possibility.

The current study was designed to test the hypothesis that the facilitation of the category specific, item general cognitive operations is not dependent upon the continued availability of the category label in declarative memory. This experiment extends the work of Woltz and Was (2006) and Hirsch et al. (2021) in several ways. First, the current experiment directly tested the premise that procedural memory is facilitated by the explicit categorization of items into a category with the category label being explicitly presented. In these previous studies, participants were indirectly told to categorize (e.g., remember the trees, ignore the relatives) during a memory load of exemplars. In the current experiment, participants directly categorized exemplars into one of four explicitly labeled categories. Second, the current experiment examines if procedural memory can be facilitated for specific categories in the absence of declarative memory recall of those very same category labels. Additionally, in the present work the demonstration of lack of declarative memory maintenance (i.e., inability to recall category labels) occurred before demonstration of the facilitation of procedural memory (i.e., category comparisons).

## Method

### Participants

138 undergraduates at a large Midwestern university participated in exchange for course credit. Two participants were dropped from the analyses because of missing data due to computer malfunctions. The final sample size was 136 participants. Median age of the sample was 23 years (range 19–49). To determine sample size, we conducted an a priori power analysis using G*Power (Faul et al., 2007) with a η^2^ = .10, α = .05, β = .80. We chose this effect size to be conservative as the smallest η^2^ reported by Woltz and Was (2006) was .14. The power analysis suggested an *N* = 90. Our stopping rule was based on the end of the semester research credit sign ups.

### Apparatus

The participants performed the experimental task in groups of 1–6. The experimental task was administered on IBM-compatible microcomputers with SVGA monitors and standard keyboards. Each computer was in a separate carrel within the laboratory. The experiment was programmed with E-Prime software (Psychology Software Tools, 2020).

### Procedure

Participants completed one of three versions of the following task. Across these three versions, categories and exemplars used for primed and unprimed categories were counterbalanced across participants (see Appendix). A block consisted of each component of the task in the following order: categorization, filler, category recall, category comparisons. A practice block containing accuracy and response time feedback preceded 11 experimental blocks without feedback.

#### Categorization

Participants were given the following categorization instructions: “*Get ready to categorize words. Place your fingers on the 1, 2, 3, and 4 keys; you'll use these to respond. Here are the current categories:”* Categories were displayed below these instructions from 1 (top) to 4 (bottom) and remained on the screen throughout this component of the task. Once participants pressed the Space Bar to advance, a fixation point (*) replaced the instructions and was shown for 1000 ms. An exemplar of one of the categories then replaced the fixation point and remained on the screen for up to 5000 ms or until the participant pressed the 1, 2, 3, or 4 key. The fixation points and exemplars were repeated until two exemplars from each of the four categories had been displayed and categorized. All text was presented in size 18 Courier New Font. This component of the task was intended to facilitate the cognitive procedure of categorizing an exemplar in particular categories.

#### Filler

Participants next completed the Digit Recoding Task, a content-embedded working memory task (Was et al., 2011), as a filler task. This task was selected to prevent participant rehearsal of category labels prior to recall. As participants would have to maintain five numbers in mind and answer two questions about their ordering or relationships, it was presumed this would tax their working memory sufficiently to preclude category label rehearsal.

Participants were given the following instructions: “*You will next be presented with five numbers that you'll have to remember. You will then be given instructions for what to do with these numbers while you keep them in mind. For example, if you were shown "4 6 1 8 9" and asked: ‘What number follows 1?’, you would answer ‘8’.”* Digits were presented individually for 2225 ms followed by a 500 ms fixation point (*). Participants were then given a question regarding the ordering or relationship among the presented numbers that was displayed until participants pressed a number key to indicate an answer. All text was presented in size 18 Courier New font.

#### Category Recall

Participants next attempted to recall the four category labels they had used in the most recent categorization component of the task. Participants were given the following instructions: “*Previously, you saw words that you assigned to categories. Your task now is to recall the categories. During recall, type in all the categories you can remember. Don’t type in the words you categorized, just the categories. Press ‘9’ when finished*.” Participants then advanced to a recall screen that instructed “*Type in the categories you most recently saw. Press ‘9’ when finished*” at the top of the screen and were given unlimited time to recall the four most recent category labels from the current block’s categorization component. The words that participants typed appeared under these instructions. The dependent variable for this component of the task was the accuracy of category label recall of the four categories used in the current block’s categorization component. All text was presented in size 14 or 16 Courier New font.

#### Category Comparisons

This component of the task required participants to make like/different judgments for category exemplars. This task component has been used extensively in previous work demonstrating the facilitation of procedural memory (Was & Woltz, 2007; Woltz & Was, 2006, 2007). Categories were either primed (i.e., used in the current block’s categorization component) or unprimed (i.e., not used elsewhere in the task). Two exemplars were presented at a time and there were 12 comparisons total. Of the comparisons, four were “like” judgments from the four primed categories (e.g., “daughter, uncle” if relative was a primed category), four were “different” judgments from the four primed categories (e.g., “nephew, Labrador” if relative was a primed category), two were “like” judgments from unprimed categories, and two were “different” judgments from unprimed categories. Importantly, exemplars used in comparison judgments were not used elsewhere in the task, meaning that comparisons for primed categories did not feature exemplars used in the categorization component of the task.

Participants were told that the term “like” meant that the two items presented were from the same category and that “different” meant the two items came from different categories. Participants were given the following instructions regarding the category comparisons: “*Your next task will now be to compare words. Place your fingers on the ‘L’ and ‘D’ keys. Press ‘L’ for ‘like’ words. Press ‘D’ for ‘different’ words. The task will begin automatically.”* This screen displayed for 10,000 ms followed by a blank screen for 500 ms, followed by alternations between a fixation point (*) displayed for 1000 ms and the category comparison screen displayed for 5000 ms (unless answered sooner). Throughout both fixation and comparison screens, “D = Different; L = Like” were displayed on the bottom of the screens. Category exemplars appeared at the top of the screen during comparisons. All text was written in Courier New font; instructions were size 16, “D = Different; L = Like” were size 14, and category exemplars were size 20. Initial dependent variables were comparison judgment accuracy and response time (RT) in ms for each primed-like comparison and unprimed-like comparison in each block. Participant accuracy and RT were then averaged over blocks before being converted into a speed score that represented correct answers per minute (i.e., mean response accuracy / mean RT x 60,000). Greater speed scores for primed categories compared to unprimed categories would indicate facilitation of procedural memory.

## Results

Participants’ mean accuracy for the categorization task was .92 (*SD* = .05). Based on the descriptive statistics it is apparent that participants accurately categorized the exemplars during the categorization task. Regarding the filler task, participants performed with relative accuracy (*M* = .64, *SD* = .16). Although this is lower than is typical of performance on this task (e.g., Was et al., 2011), this is likely due to two outliers with accuracy scores of .14. These participants were not withdrawn from remaining analyses because their categorization task scores were close to the mean or well above chance (.94 and .75 respectively). Mean accuracy for category recall was .76 (*SD =* .19) suggesting that participants were moderately successful at recalling the categories, yet with some inaccurate or unsuccessful recall.

The hypotheses regarding response facilitation were tested with a dependent variable created by transforming and combining response time and response accuracy variables. Because facilitation effects are typically evident in both latency and accuracy measures in previous research (e.g. Woltz & Was, 2006, 2007), a combined measure was computed for each trial condition as the participant’s number correct for comparisons in that condition divided by the sum of response latency for all the comparisons in that condition (both correct and incorrect). This measure represents an index of response speed adjusted for errors: It is the reciprocal of response latency, and it adjusts speed according to errors. It can be interpreted directly as number of correct responses per unit time (minutes in this case) and is referred to as rate correct score (RCS). This index has the advantage of incorporating meaningful variance of both latency and error rates, and the distribution usually approximates the normal distribution more closely than does that of either the latency or the error distribution. All statistical tests corresponding to our hypotheses were conducted using this dependent variable. Only data from positive match comparisons were analyzed, on the basis of prior evidence that priming effects such as these are negligible in negative match comparisons (Woltz & Was, 2006).

**Figure 1:**
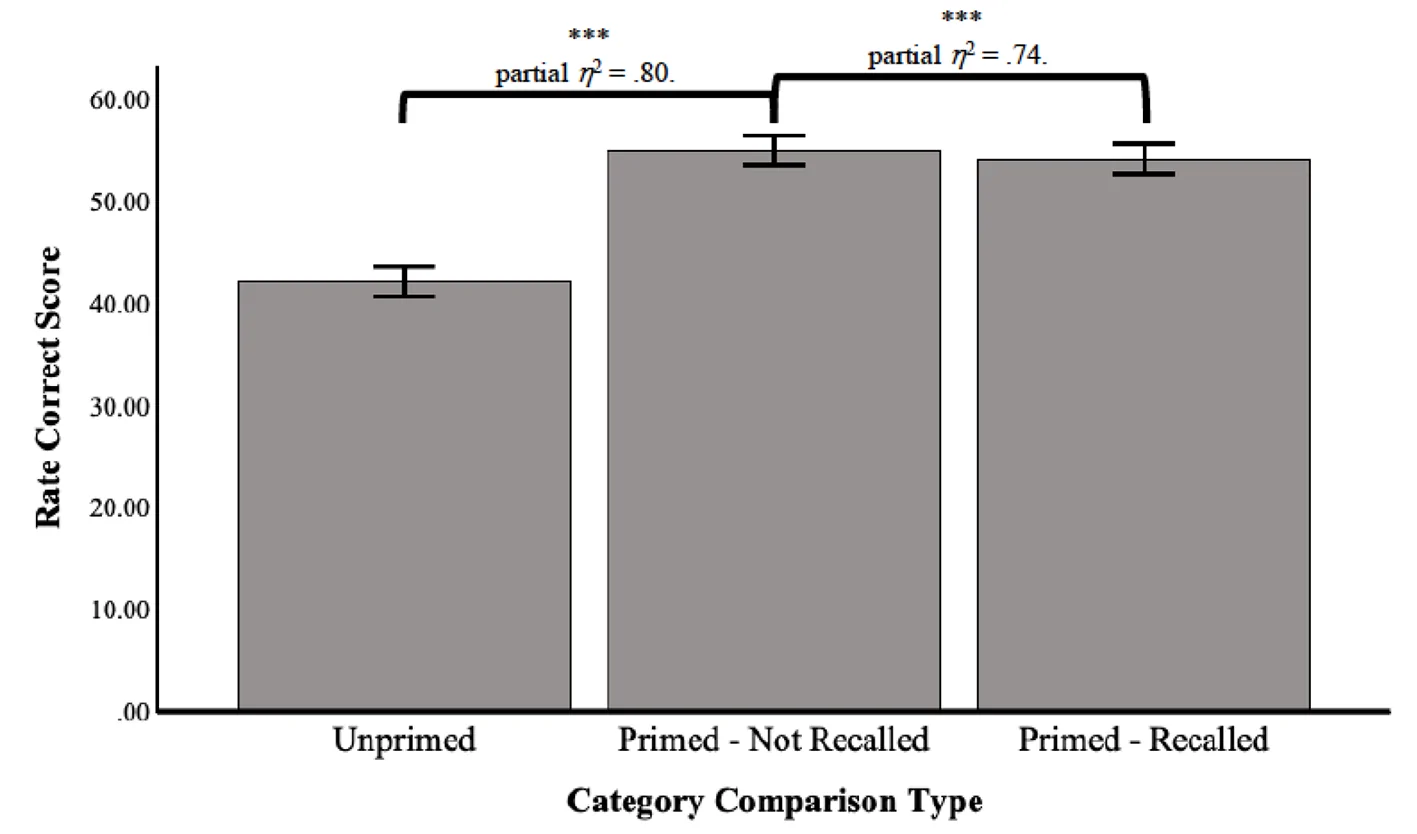
Rate Correct Score for Positive Match Category Comparison Trials by Category Type ***p < .001.

To test our hypothesis that procedural memory can be facilitated for specific categories in the absence of declarative memory recall of those very same category labels we created two dependent variables from primed categories. Recalled category comparisons are category comparisons for which the participant was able to accurately recall the category label. Non-recalled category comparisons are comparisons for which the individual participant was unable to accurately recall the category label. We then contrasted RCS for these two conditions with category comparison RCS for unprimed categories. The mean latency and error data from which the speed scores were computed are reported in Table 1. Figure 1 presents the mean RCS data for positive match category comparisons by trial condition.

**Table 1:** Means and Standard Deviations of Category Comparison Response Times and Accuracy by Comparison Type

	Response Time (ms)		Accuracy (Proportion Correct)	
	Mean	*SD*	Mean	*SD*
Unprimed	1306.58	268.96	.87	.10
Primed – Not Recalled	1279.61	253.58	.90	.08
Primed - Recalled	1297.64	260.03	.90	.08

To test the hypothesis that the facilitation of the category specific, item general cognitive operations is not dependent upon the continued availability of the category label in declarative memory we conducted a within-subjects analysis (repeated measures ANOVA) with planned comparisons. The comparisons allowed us to compare response speed of the three different category comparison trial types: Unprimed (i.e., not previously processed) category comparisons, with primed comparisons (previously processed) of which the participant did not recall the category, and primed comparisons of which the participant did not recall the category with those category comparisons that they did recall the category. The repeated measures ANOVA revealed that the main effect of category comparison type was significant, *F*(2, 135) = 501.47 *p* < . 001, η^2^ = .79. Planned comparisons indicated that participants responded more quickly and accurately during the category comparisons to novel exemplar comparisons of non-recalled primed categories (*M =* 54.87, *SD =* 8.76) than novel exemplar comparisons from unprimed categories (*M =* 42.01, *SD =* 8.78), *F* (1, 135) = 542.95, *p < .*001, partial η^2^ = .80. Interestingly, participants more quickly and accurately categorized novel exemplars from non-recalled categories compared to novel exemplars from recalled categories (*M =* 54.02, *SD =* 8.55), *F* (1, 135) = 384.54, *p < .*001, partial η^2^ = .74.

The counterintuitive result that non-recalled categories showed greater facilitation than recalled ones might be driven by specific subgroups rather than representing a universal pattern. Put differently, it could be that individual differences, such as strategic approaches to the task, might have resulted in different patterns of results during the category comparison task. Thus, we chose to further analyze the data by first examining the correlation between category recall accuracy and category comparison RCS, and then grouping participants based on median recall accuracy and again running the repeated measures ANOVA. The correlation between category recall accuracy and primed category comparison RCS was not significant, *r* = .16, *p* = .07. A 2 (above median recall accuracy v. below median recall accuracy) x 3 (category comparisons: unprimed, unrecalled primed, recalled primed) again indicated significant priming effects but no effect of median split group, *F* < 1, nor was there a significant interaction, *F* (1, 134) = 1.64, *p* = .20.

**Figure 2:**
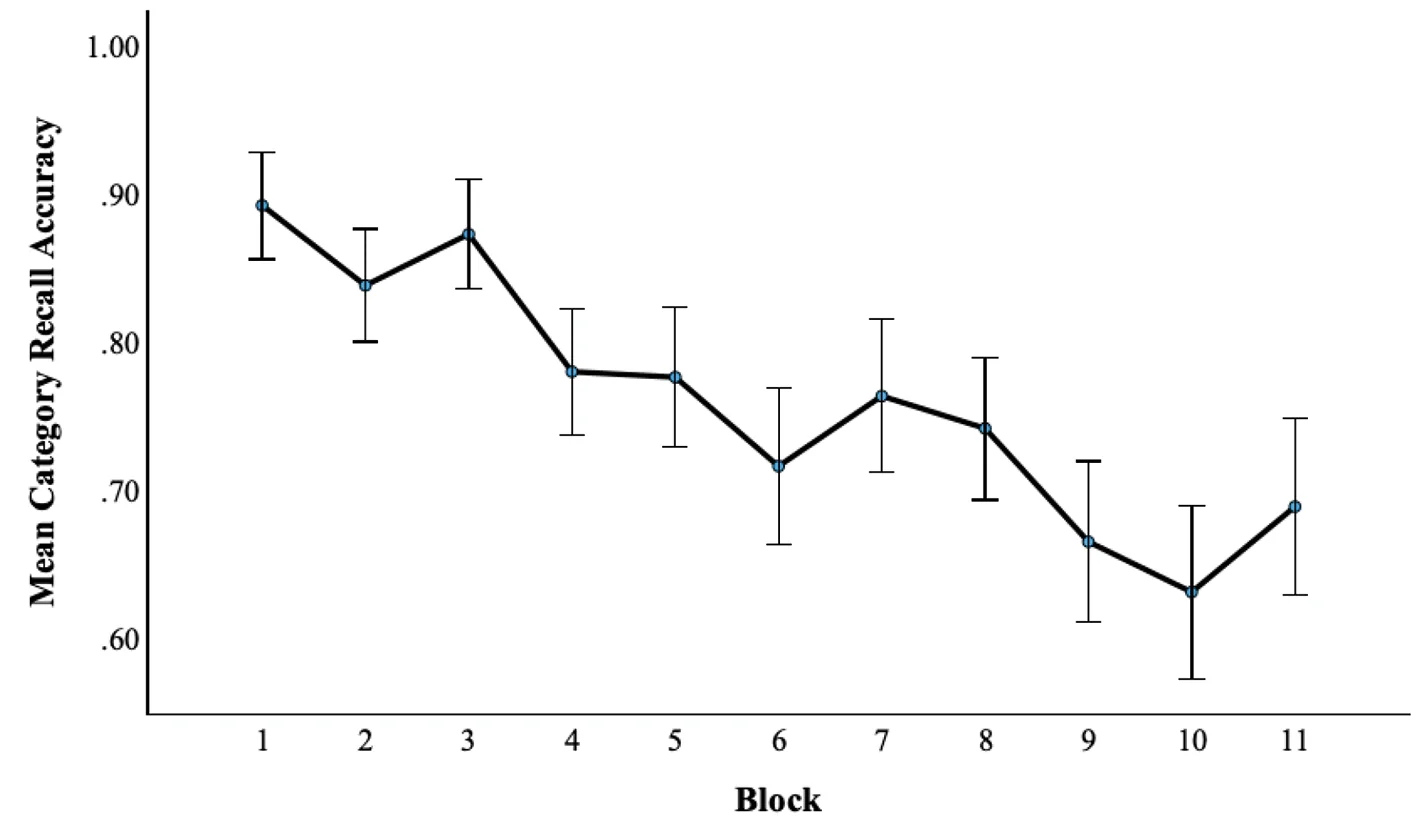
Mean Category Recall Accuracy by Block

To further test the dissociation between declarative memory maintenance and the facilitation of procedural memory we examined response trends across time (blocks of trials) of both category recall accuracy and speed of response to all primed (primed unrecalled and primed recall) category comparisons. We first conducted a repeated measures ANOVA with simple contrasts comparing the first block of category recall to subsequent blocks. Figure 2 displays mean category recall accuracy across blocks. The between subjects effects were significant *F* (10, 136) = 18.45, *p <* .001, partial η^2^ = .12 and the within subjects contrast between block 1 and block 2 as significant *F* (1, 136) = 6.54, *p <* .012, partial η^2^ = .05 as was the contrast between block 1 and block 11, *F* (1, 136) = 38.00, *p <* .001, partial η^2^ = .22. The results indicate that participants’ recall of categories declined over the blocks of trials. Whether this represents an effect of fatigue or proactive interference is open for debate, but it does suggest that participants were less able to maintain accurate declarative representations of the categories as the task progressed.

**Figure 3:**
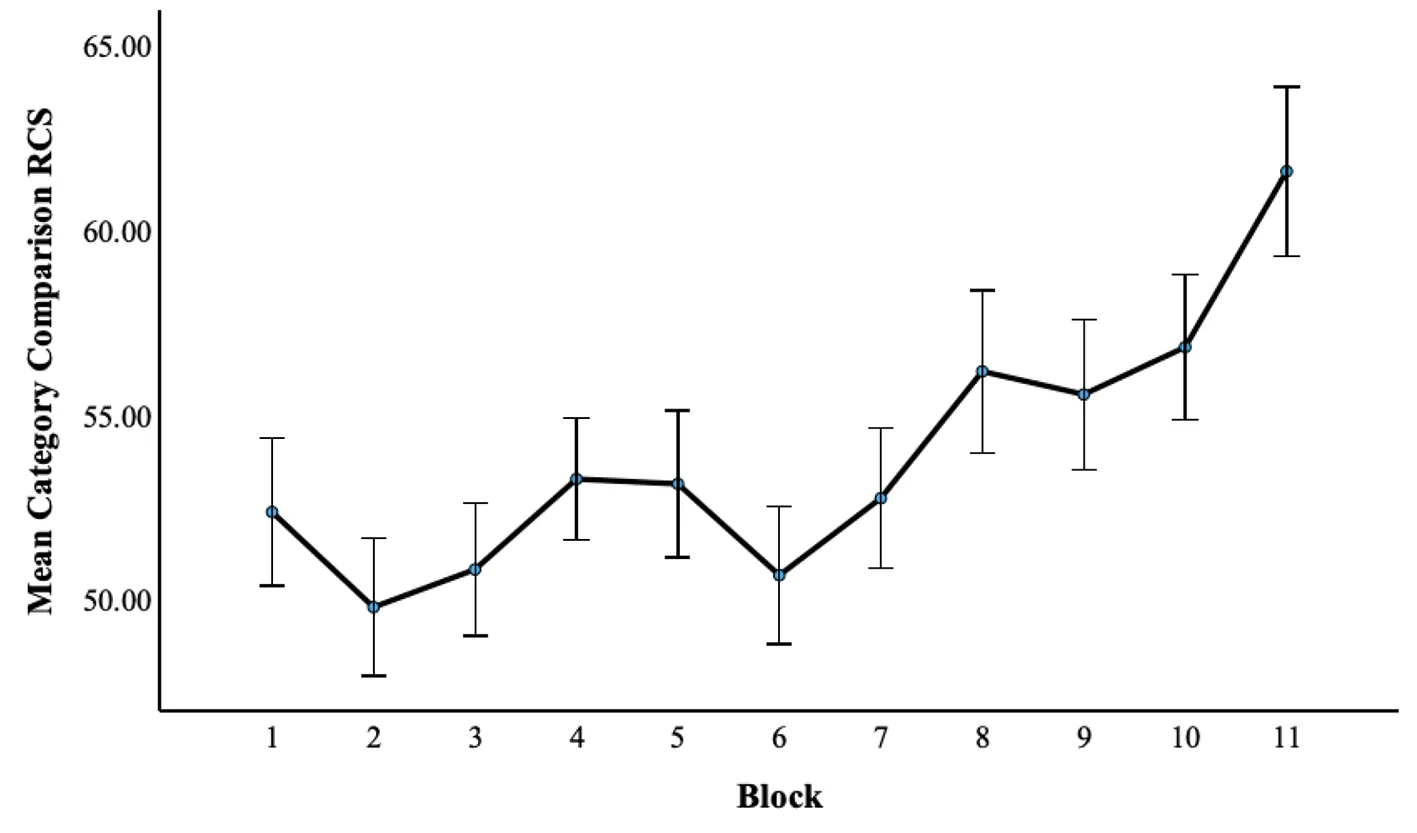
Mean Category Comparison RCS by Block

Next, we conducted a repeated measures ANOVA with simple contrasts comparing the first block of all primed category comparison to subsequent blocks. Figure 3 displays mean category comparison RCS across blocks. The between subjects effects were significant *F* (10, 135) = 24.48, *p <* .001, partial η^2^ = .15 and the within subjects contrast between block 1 and block 2 was significant, *F* (1, 135) = 9.99, *p <* .002, partial η^2^ = .07, as was the contrast between block 1 and block 11, *F* (1, 135) = 67.87, *p <* .001, partial η^2^ = .34. The results indicate that participants’ RCS increased over the blocks of trials. These results suggest that participants were faster and more accurate when making primed category comparisons. This may in part represent a practice effect, but it is consistent with the conclusion that participants continue to experience the facilitation of procedural memory over the course of the experiment.

Thus, the results suggest that the facilitation of procedural memory (i.e., faster and more accurate categorization of exemplars of specific, practiced categories) is not dependent upon declarative memory maintenance (i.e., maintaining access to specific, practiced category labels).

## Discussion

The current study addressed two research questions. First, does the facilitation of procedural memory result from explicit declarative categorization with the category label present? Second, does the facilitation of procedural memory occur even for categories whose label is not declaratively maintained? Analyses targeting the first research question demonstrated another replication and extension of the facilitation of procedural memory (Woltz & Was, 2006). Here, the facilitation of procedural memory was observed in a different design than the typical memory load task. In the present experiment, participants explicitly categorized exemplars of categories into categories whose labels were directly presented to participants. Later comparison of unpresented category exemplars from these practiced categories were significantly facilitated (i.e., more correct responses per minute) compared to comparison of unpresented category exemplars from non-practiced categories. These comparisons were thought to require the same cognitive procedure (e.g., “*is X a [category label]?”*) used during the previous explicit declarative categorization.

Regarding the second research question, the facilitation of procedural memory was observed for categories whose labels could not be recalled. In line with Hirsch et al. (2021), this suggests a dissociation between declarative and procedural memory. In other words, procedural operations were made faster and more accurate following similar prior procedural operations and did not rely on the continued declarative memory maintenance of the category labels. Both practiced categories that were explicitly recalled and practiced categories that were not explicitly recalled were facilitated compared to non-practiced categories. Most interestingly, the facilitation of procedural memory was greatest for practiced categories that were not explicitly recalled. In fact, there was significantly greater facilitation for non-recalled practiced categories compared to recalled practiced categories.

The present results are consistent with Experiment 3 of Hirsch et al. (2021), where a dissociation between declarative memory and procedural memory was also observed. In Hirsch et al., participants directed to forget category labels were unable to explicitly recognize these category labels later, despite showing facilitated performance for novel exemplar comparisons that required the cognitive procedure of categorizing within these categories. In the current experiment, participants unable to explicitly recall recently practiced category labels still showed facilitated performance for novel exemplar comparisons that required the cognitive procedure of categorizing within these categories. Taken together, along with Woltz and Was (2006), the results suggest that the facilitation of procedural memory is most likely based on the strengthened cognitive procedure of categorizing and not the active maintenance of the declarative category label, exemplars, or semantic priming.

These findings are more broadly consistent with previous reports of dissociations between declarative and procedural memory abilities in neuropsychological (e.g., Squire & Zola, 1996) and neuroimaging research (Poldrack et al., 2001). Indeed, there is abundant evidence that these declarative and procedural memory processes are associated with mostly distinct neuroanatomical substrates. Specifically, declarative memory relies on the hippocampus and the surrounding medial temporal lobe structures, while procedural memory relies on the basal ganglia, in particular the striatum, and possibly some neocortical structures and the cerebellum (e.g., Ullman, 2016). Although these systems are dissociable, they may interact cooperatively or competitively (Hamrick et al., 2018; Poldrack & Packard, 2003).

The findings also fit well within the ACT-R framework. Recall that in Anderson’s (2009) ACT-R model of cognition goal states and active declarative memory elements trigger operations of procedural memory. These operations then produce appropriate responses to move towards the goal state. Once procedural memory performs its operations, new declarative memory elements are activated, and the process repeats until the goal state is attained. It could be that as new goal states—in this case *correctly identify the category in which the current exemplar belongs*—activates new declarative memory elements while the cognitive operation remains facilitated.

Furthermore, the findings align with a foundational assumption regarding the distinction between procedural memory and the semantic components of declarative memory, as outlined by Anderson’s (2009) theory. In contrast to the temporary activation of declarative structures, memory for the cognitive operations performed on the stimuli are assumed to be more persistent. In the present example, this includes memory for the encoding operations and, more importantly, the category identification operations. A subsequent exemplar comparison that asks whether maple and elm are from the same category shows stronger facilitation because memory representations for both the encoding and category identification operations were recently strengthened.

The finding in the current experiment of a dissociation between declarative and procedural memory (i.e., facilitated procedural memory in the absence of declarative memory recall) also aligns with Oberauer’s (2009) distinction between declarative and procedural working memories. However, the finding in the current experiment of greater facilitation for non-recalled categories does not easily integrate into Oberauer’s model. For example, according to Oberauer’s model in a simple parity task (i.e., decide if a number is even or odd) it is presumed that the bridge (i.e., procedural working memory) holds a task set and response (e.g., if number is odd, press the “o” key). It seems necessary that the declarative categories of “odd” and “even” must be held somewhere in procedural working memory to be maintained in the bridge to complete the task. Procedural operations are facilitated if the current response is subsequently executed, as the task set and response mappings are already in the bridge. In the current experiments, participants demonstrated facilitated procedural memory (e.g., facilitated bridge functioning) despite not being able to recall the declarative category labels that were facilitated. This suggests that the current facilitated category labels were not maintained, conflicting with the proposition in Oberauer’s (2009) model that category labels used in procedural operations are maintained in the bridge. Given the novelty of the finding of increased facilitation for non-recalled categories compared to recalled categories, this finding should be replicated before any strong conclusions regarding model and theory fit are warranted.

### Contributions and Future Directions

The current research contributes to the facilitation of procedural memory literature and sets up interesting work for the future. First, the current work provided another replication of the facilitation of procedural memory as well as an extension of this work. Replication is critical to sound science and reliable conclusions (McIntyre, 2019) and the facilitation of procedural memory was replicated using a similar but novel categorization practice task.

The current research also provided a replication and extension of related work demonstrating a dissociation between declarative and procedural memory. Similar to Hirsch et al. (2021), the current research demonstrated a lack of declarative activation and access despite facilitated procedural memory operations. In Hirsch et al., participants were directed to forget category labels and were ultimately unable to recognize these category labels despite showing facilitated categorization within these categories (Experiment 3). Similar findings were obtained in the current research using a category label recall task; here, participants who were unable to recall recently used category labels still showed facilitated categorization within these categories.

An alternative interpretation of the current findings is that there was not a strong dissociation between declarative and procedural memory, but rather a gradient of activation and conscious accessibility. For example, it is possible that recalling or attempting recall of category labels increased activation of these categories and related information, which was responsible for the facilitated performance observed on subsequent category comparisons. Even non-recalled categories could be facilitated as attempted recall could increase activation (albeit below recall threshold) which could support facilitated category comparisons. This possibility is described in Cowan’s embedded processes model of working memory (1999, 2005), where elements from activated long-term memory can be readily drawn into the focus of attention (i.e., conscious awareness) despite not being currently activated to the threshold of conscious awareness. However, this explanation does not account for the increased facilitation of non-recalled categories compared to recalled categories. In the alternative gradient explanation, recalled categories should show the highest level of facilitation due to the higher level of activation; non-recalled categories should show more facilitation than unprimed categories but less activation than recalled categories due to their higher-than-baseline but lower-than-recalled-categories’ activation. As the alternative explanation for the observed dissociation between declarative and procedural memory cannot account for this finding, the proposed explanation of greater facilitation due to prior procedural practice better fits the data.

This finding of greater facilitation for non-recalled categories is interesting but should be replicated before strong conclusions are made. While it is possible that the finding of greater facilitation for non-recalled categories is a false positive, prior research suggests that this type of facilitation is likely to be a true effect. Woltz and Was (2006) and Hirsch et al. (2021) have demonstrated that ignored or forgotten categories show as much or greater facilitation than categories requiring some form of declarative memory maintenance. If future research can replicate this effect, it would then be interesting to uncover the mechanism behind greater facilitation for non-recalled categories.

## Supplementary Materials

Supplementary materials include the category and exemplar stimuli used in the task. We have also included the raw data in the supplementary materials (see Was, 2024).



WasC. A.
 (2024). The facilitation of cognitive procedures is not dependent on maintenance of declarative memory elements
[Data, materials]. PsychOpen. https://osf.io/phw7f


## Data Availability

For this article, data is freely available (see Was, 2024).
